# Supplementary material for: Remote control of AMPK via extracellular adenosine controls tissue growth
Source: Nat Cell Biol. 2025 Sep 26;27(10):1827–37. doi: 10.1038/s41556-025-01764-0 (PMC12527939; doi:10.1038/s41556-025-01764-0)
Supplement: Supplementary file 1 — Reporting Summary [file 41556_2025_1764_MOESM1_ESM.pdf]

nature portfolio

Corresponding author(s): Aurelio Teleman

Last updated by author(s): Jul 16, 2025

Reporting Summary

Nature Portfolio wishes to improve the reproducibility of the work that we publish. This form provides structure for consistency and transparency in reporting. For further information on Nature Portfolio policies, see our Editorial Policies and the Editorial Policy Checklist.

Statistics

For all statistical analyses, confirm that the following items are present in the figure legend, table legend, main text, or Methods section.

n/a	Confirmed	
☐	☒	The exact sample size (*n*) for each experimental group/condition, given as a discrete number and unit of measurement
☐	☒	A statement on whether measurements were taken from distinct samples or whether the same sample was measured repeatedly
☐	☒	The statistical test(s) used AND whether they are one- or two-sided<br>*Only common tests should be described solely by name; describe more complex techniques in the Methods section.*
☒	☐	A description of all covariates tested
☐	☒	A description of any assumptions or corrections, such as tests of normality and adjustment for multiple comparisons
☐	☒	A full description of the statistical parameters including central tendency (e.g. means) or other basic estimates (e.g. regression coefficient) AND variation (e.g. standard deviation) or associated estimates of uncertainty (e.g. confidence intervals)
☐	☒	For null hypothesis testing, the test statistic (e.g. *F*, *t*, *r*) with confidence intervals, effect sizes, degrees of freedom and *P* value noted<br>*Give P values as exact values whenever suitable.*
☒	☐	For Bayesian analysis, information on the choice of priors and Markov chain Monte Carlo settings
☒	☐	For hierarchical and complex designs, identification of the appropriate level for tests and full reporting of outcomes
☒	☐	Estimates of effect sizes (e.g. Cohen's *d*, Pearson's *r*), indicating how they were calculated

Our web collection on statistics for biologists contains articles on many of the points above.

Software and code

Policy information about availability of computer code

Data collection	Leica confocal microscope SP8 with software Leica Application Suite X (LAS X) version 3.5.2.18963 was used.
Data analysis	All software is publicly or commercially available:<br>Images analysis: ImageJ/FIJI v 2.14.0.<br>RNA-seq analysis: the galaxy server platform (https://usegalaxy.org)<br>GO enrichment analysis: the online tool http://www.webgestalt.org.<br>Figure preparation: Affinity Photo v1.<br>Data analysis: Microsoft Excel v16 or Graphpad Prism v10.5

For manuscripts utilizing custom algorithms or software that are central to the research but not yet described in published literature, software must be made available to editors and reviewers. We strongly encourage code deposition in a community repository (e.g. GitHub). See the Nature Portfolio guidelines for submitting code & software for further information.

Data

Policy information about availability of data

All manuscripts must include a data availability statement. This statement should provide the following information, where applicable:

- Accession codes, unique identifiers, or web links for publicly available datasets
- A description of any restrictions on data availability
- For clinical datasets or third party data, please ensure that the statement adheres to our policy

RNA-seq data are deposited at NCBI with accession GSE284402 and raw counts are provided in Suppl. Table 4. All other data are available in the main text or the supplementary materials.

Research involving human participants, their data, or biological material

Policy information about studies with human participants or human data. See also policy information about sex, gender (identity/presentation), and sexual orientation and race, ethnicity and racism.

Reporting on sex and gender	Not applicable.
Reporting on race, ethnicity, or other socially relevant groupings	Not applicable.
Population characteristics	Not applicable.
Recruitment	Not applicable.
Ethics oversight	Not applicable.

Note that full information on the approval of the study protocol must also be provided in the manuscript.

Field-specific reporting

Please select the one below that is the best fit for your research. If you are not sure, read the appropriate sections before making your selection.

☒ Life sciences ☐ Behavioural & social sciences ☐ Ecological, evolutionary & environmental sciences

For a reference copy of the document with all sections, see nature.com/documents/nr-reporting-summary-flat.pdf

Life sciences study design

All studies must disclose on these points even when the disclosure is negative.

Sample size	No sample size calculations were performed. Sample sizes were determined based on practical considerations. Based on these considerations, sample sizes were set at >8 wing discs per genotype/condition, which allowed modest standard deviations and significant p values to be obtained.
Data exclusions	No data were excluded.
Replication	The number of replicates is indicated in the figure legends.
Randomization	All animals were randomly alloted.
Blinding	The experiments were not blinded because they each contained an internal control and because blinding is not usual in this field.

Reporting for specific materials, systems and methods

We require information from authors about some types of materials, experimental systems and methods used in many studies. Here, indicate whether each material, system or method listed is relevant to your study. If you are not sure if a list item applies to your research, read the appropriate section before selecting a response.

Materials & experimental systems

n/a	Involved in the study
☐	☒ Antibodies
☒	☐ Eukaryotic cell lines
☒	☐ Palaeontology and archaeology
☐	☒ Animals and other organisms
☒	☐ Clinical data
☒	☐ Dual use research of concern
☒	☐ Plants

Methods

n/a	Involved in the study
☒	☐ ChIP-seq
☒	☐ Flow cytometry
☒	☐ MRI-based neuroimaging

Antibodies

Antibodies used	rabbit anti phospho-AMPKa(T172) (Cell signaling 50081), rabbit anti phospho-S6K (T398) (PhosphoSolutions p1705-398) 1:1000, rabbit anti-AMPKa (Cell signaling 2532S) 1:1000, rabbit anti-tubulin (Cell signaling 2125) 1:1000, Guinea pig anti Drosophila S6K (1:2000), rabbit anti phospho-ribosomal protein S6 1:1000
Validation	rabbit anti phospho-ribosomal protein S6 1:1000 was validated in the publication: PMID8829945<br>Guinea pig anti Drosophila S6K (1:2000) was validated in the publication: PMID20444422<br>rabbit anti phospho-AMPKa(T172) (Cell signaling 50081) was validated in https://www.cellsignal.com/products/primary-antibodies/phospho-ampka-thr172-d4d6d-rabbit-mab/50081<br>rabbit anti-AMPKa (Cell signaling 2532S) 1:1000 was validated in https://www.cellsignal.com/products/primary-antibodies/ampka-antibody/2532<br>rabbit anti-<br>atubulin (Cell signaling 2125) 1:1000 was validated in https://www.cellsignal.com/products/primary-antibodies/a-tubulin-11h10-rabbit-mab/2125

Animals and other research organisms

Policy information about studies involving animals; ARRIVE guidelines recommended for reporting animal research, and Sex and Gender in Research

Laboratory animals	Species: Drosophila melanogaster<br>Strains: Full genotype for each figure panel is provided in Suppl. Table 3.
Wild animals	No wild animals were used.
Reporting on sex	Sex was generally not considered in the study design
Field-collected samples	This study did not involve samples collected from the field.
Ethics oversight	This study does not require an ethical approval.

Note that full information on the approval of the study protocol must also be provided in the manuscript.

Plants

Seed stocks	Not applicable.
Novel plant genotypes	Not applicable.
Authentication	Not applicable.
