# Supplementary figures and images for: Remote control of AMPK via extracellular adenosine controls tissue growth
Source: Nat Cell Biol. 2025 Sep 26;27(10):1827–37. doi: 10.1038/s41556-025-01764-0 (PMC12527939; doi:10.1038/s41556-025-01764-0)

**Fig. 4a**

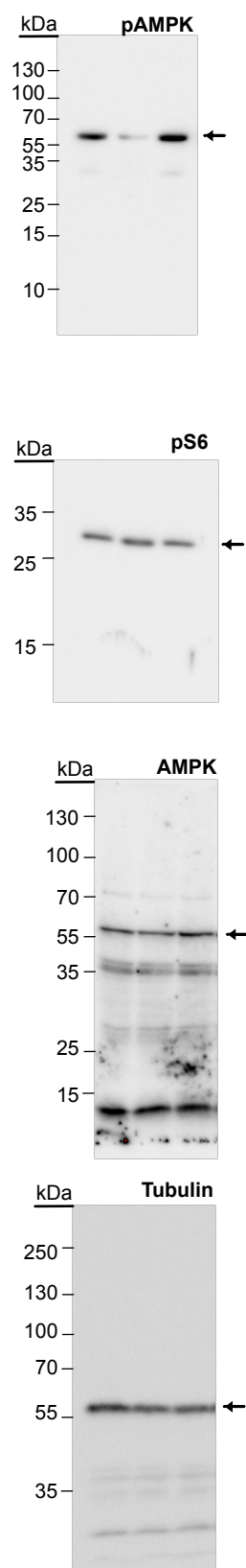

**Fig. 4b**

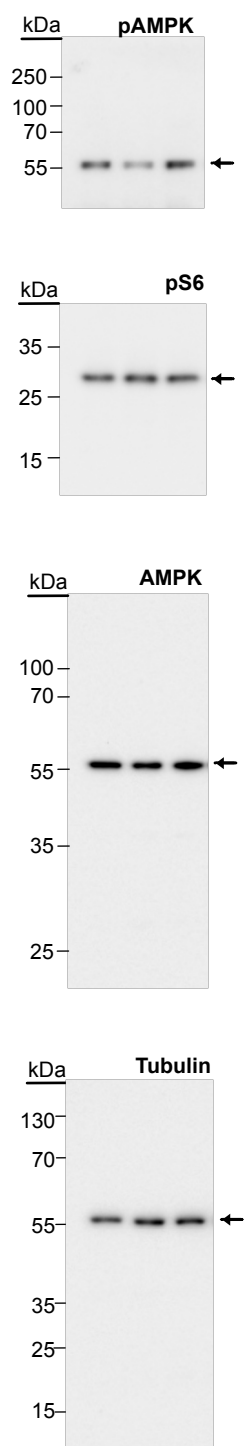

**Fig. 4f**

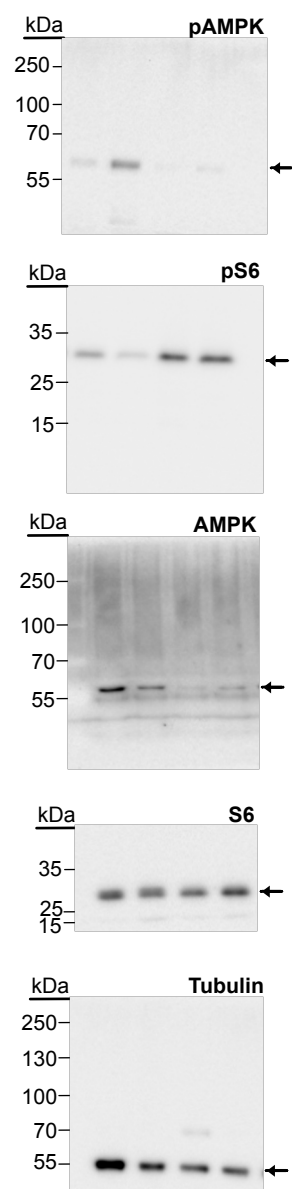

### ED Fig. 4c

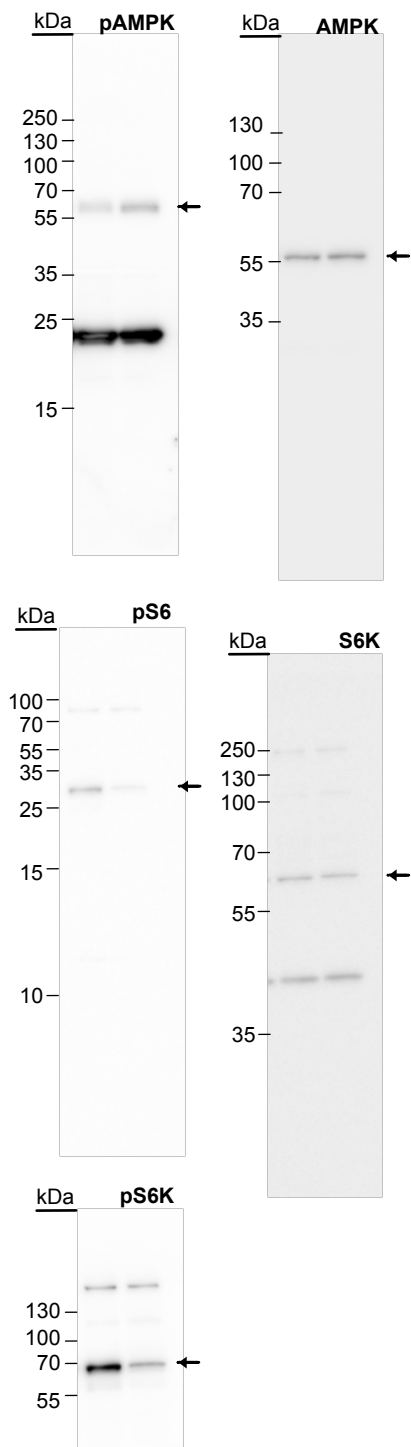

### ED Fig. 4d

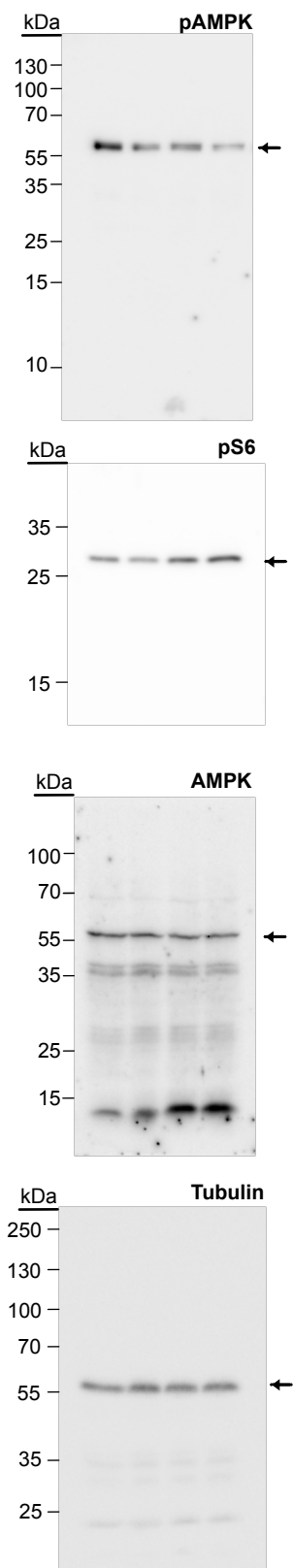

### ED Fig. 4e

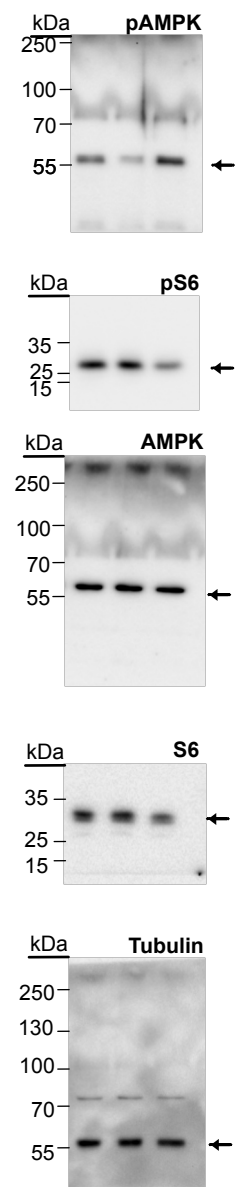

## ED Fig. 4f

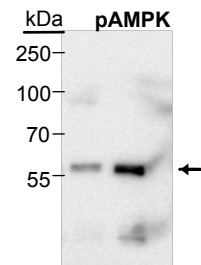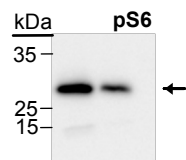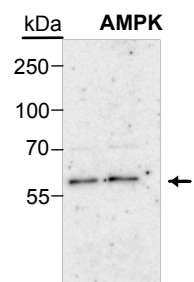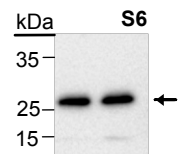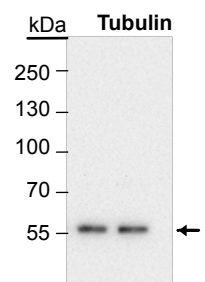

**ED Fig. 7a**

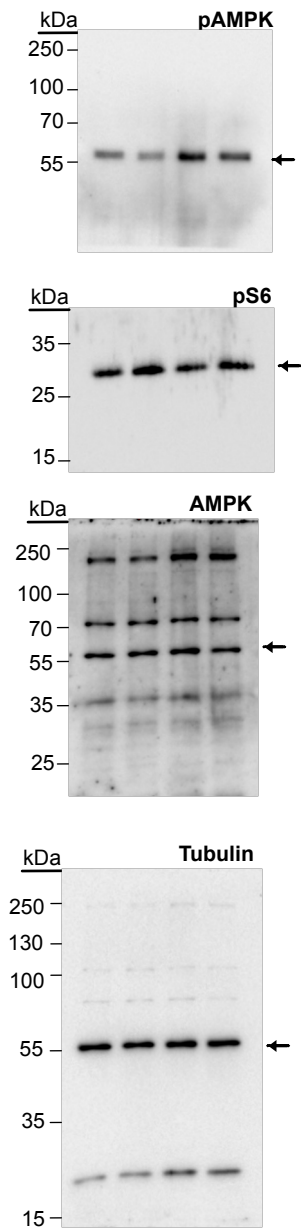

**ED Fig. 7b**

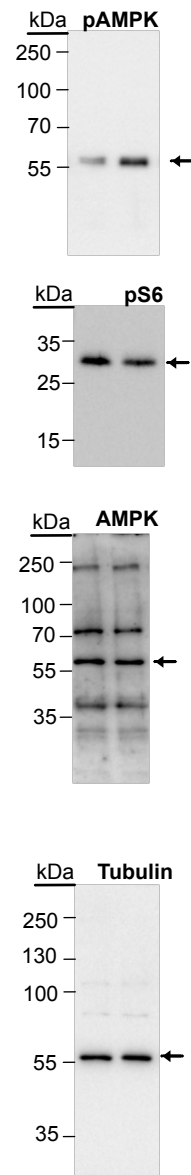

Supplement: Supplementary file 5 — Unprocessed western blots. [file 41556_2025_1764_MOESM5_ESM.pdf]
